# Health-related quality of life worsens by school age amongst children with food allergy

**DOI:** 10.1186/s13601-019-0244-0

**Published:** 2019-02-07

**Authors:** Victoria Thörnqvist, Roelinde Middelveld, Hay Mar Wai, Natalia Ballardini, Evalill Nilsson, Jennie Strömquist, Staffan Ahlstedt, Lennart Jan Nilsson, Jennifer L. P. Protudjer

**Affiliations:** 1 The Centre for Allergy Research, Karolinska Institutet, Box 210, 171 77 Stockholm, Sweden; 2 The Institute of Environmental Medicine, Karolinska Institutet, Stockholm, Sweden; 3 Sachs’ Children and Youth Hospital, Sodersjukhuset, Stockholm, Sweden; 4 Pediatric Allergy, Linköpings Universitetet, Linköping, Sweden; 5 Department of Medical and Health Sciences, Linköping University, Linköping, Sweden; 6 Department of Pediatrics and Child Health, University of Manitoba, Winnipeg, Canada; 7 George and Fay Yee Centre for Healthcare Innovation, Winnipeg, Canada; 8 The Children’s Health Research Institute of Manitoba, Winnipeg, Canada

**Keywords:** Children, Food allergy, Food hypersensitivity, Health-related quality of life

## Abstract

**Background:**

Food allergy is negatively associated with health-related quality of life (HRQL). Although differences exist between parents and children, less is known about age-specific differences amongst children. As such, we aimed to identify if age, as well as other factors, are associated with food allergy-specific HRQL in an objectively defined population of children.

**Methods:**

Overall, 63 children (boys: n = 36; 57.1%) with specialist-diagnosed food allergy to 1 + foods were included. Parents/guardians completed the Swedish version of a disease-specific questionnaire designed to assess overall- and domain-specific HRQL. Descriptive statistics and linear regression were used.

**Results:**

The most common food allergy was hen’s egg (n = 40/63; 63.5%). Most children had more than one food allergy (n = 48; 76.2%). Nearly all had experienced mild symptoms (e.g. skin; n = 56/63; 94.9%), and more than half had severe symptoms (e.g. respiratory; 39/63; 66.1%). Compared to young children (0–5 years), older children (6–12 years) had worse HRQL (e.g. overall HRQL: B = 0.60; 95% CI 0.05–1.16; *p* < 0.04.). Similarly, multiple food allergies, and severe symptoms were significantly associated with worse HRQL (all *p* < 0.05) even in models adjusted for concomitant allergic disease. No associations were found for gender or socioeconomic status.

**Conclusion:**

Older children and those with severe food allergy have worse HRQL.

**Electronic supplementary material:**

The online version of this article (10.1186/s13601-019-0244-0) contains supplementary material, which is available to authorized users.

## Introduction

Food allergy directly affects 4–10% of children [1, 2]. To minimise the risk of reaction, food allergy demands constant vigilance around food. Such vigilance requires behavioural changes for the food allergic individual and their family. These changes and the resulting impact on the family likely change based on the child’s age and development. Elsewhere, parents reported that their child’s food allergy had less of an impact on the child’s health-related quality of life (HRQL) than the child perceived him/herself [3]. Previously, we showed that, amongst those with staple food allergy, HRQL was worse amongst those who carried an epinephrine auto-injector (EAI) or had concomitant allergic disease [4, 5]. To our knowledge, differences within an age group have not been considered. Yet, as children begin to progress through developmental stages, including gender identify, literacy and self-advocacy, prior to adolescence, we hypothesised that age may be associated with differences in food allergy-HRQL. Therefore, we undertook a cross-sectional study involving Swedish children aged 0–12 years with specialist-diagnosed food allergy with the aim of identifying if age, and other factors, are associated with food allergy-specific HRQL in amongst children with specialist-diagnosed food allergy.

## Methods

Children were recruited from two Swedish outpatient paediatric allergology clinics subsequent to a convincing history of allergy to at least one food *and* a positive Immuno-CAP test for allergen-specific Immunoglobulin E (IgE) antibodies to the same food. Exclusion criteria were an unclear food allergy diagnosis, other non-allergy chronic diseases, and/or limited understanding of the Swedish language.

### Food allergy quality of life questionnaire-parent form

Parents who provided written informed consent were asked to complete the Swedish language version of the Food Allergy Quality of Life Questionnaire-Parent Form (FAQLQ-PF). This validated questionnaire permits consideration to overall food-allergy specific HRQL, and three domains: Emotional Impact (EI), Food Anxiety (FA) and Social and Dietary Limitations (SDL; Additional file 1: Table S1) [6]. Questions which contributed to these domains are described elsewhere [4]. For each HRQL-related question, Likert 7-point scale responses ranged from “not at all” to “extremely”. From these responses, mean scores for overall- and domain-specific HRQL were calculated. Parents also reported the child’s age (dichotomised into 0–5 or 6–12 years) and household income (split at the mean of 5021€ per month; classified as “lower” or “higher.”

The FAQLQ-PF includes questions on several proxies of severity

*Previous symptoms*

Classified as “less severe” (skin, mucosal membranes, gastrointestinal, rhinoconjunctivital symptoms) and “more severe” (cardiovascular, respiratory systems).

*Anaphylaxis*

Breathing difficulties, inability to stand, collapse, loss of consciousness.

*EAI prescription*

No vs. yes.

*Concomitant allergic diseases*:

Asthma, atopic dermatitis, allergic rhinitis; classified as 0–2 vs. 3.

### Statistics

Descriptive statistics included sample sizes, means, and 95% confidence intervals (95% CI). Analytic statistics included parametric two-sample t-tests, and linear regression analyses, reported as β coefficients and 95% CI, with *p* < 0.05. Although previous research supports that gender may predict HRQL [7, 8], herein gender did not significantly alter the β coefficient and was thus excluded from further analyses. We also excluded anaphylaxis as a covariate to minimise the possibility of over-adjusting our statistical models, given the high correlation between anaphylaxis and the more rigorous and comprehensive definition of more severe symptoms. To contextualise statistical significance, minimal clinically important difference (MCID) was used (± ≥0.5 in HRQL score). Data were handled per the Swedish Personal Data Protection Act and the European Union’s General Data Protection Regulation. Ethical permission was obtained (Stockholm: DNR 2016/436-32; Linköping: DNR 2014/458-31).

## Results and discussion

Overall, 63 children, were included, of whom 36 (57.1%) were boys (Additional file 2: Figure S1). Common allergens included hen’s egg (n = 40/63; 64%), tree nut (n = 32/63; 51%) and peanut (n = 28/63; 44%; Additional file 1: Table S2). The “other” category included 8 (13%) reported allergies to seeds other than sesame seeds, rice, legumes, spices and/or poultry. Multiple food allergies were common, as were skin and respiratory symptoms. Slightly less than half of the children had previous anaphylaxis and/or an EAI prescription. Most (83%) had at least one concomitant allergic disease, and almost half (44%) had all three.

### Mean HRQL

Compared to overall HRQL, none of the domains differed significantly or achieved MCID (Additional file 1: Table S3). Compared to younger children, mean overall HRQL for older children was statistically significantly worse and almost three times greater than the MCID (3.27 vs. 2.06, respectively, *p* < 0.001; additional file 1: Table S4). Most proxies of severity were associated with significantly worse mean HRQL.

### Age

Compared to children aged 0–5 years, children aged 6–12 years had worse overall HRQL (β = 0.60, 95% CI = 0.05–1.16, *p* < 0.05), FA (β = 1.09, 95%CI = 0.50–1.67, *p* = 0.001) and EI (β = 0.66, 95% CI = 0.07–1.24, *p* = 0.03; Table 1), but not SDL, in fully adjusted models. These findings suggest that children aged 6–12 years already recognise the unique demands and stresses of being food allergic, which are reflected in the domains FA and EI. Older children go to school and other activities without parental supervision, where they are responsible for making food-related choices [9]. This is juxtaposed against cognitive immaturity and, in some cases, preliteracy. Older children may be more troubled by the ongoing need for vigilance with regard to the stresses associated with making food-related choices, rather than the avoidance mandated by their food allergies. Thus, it is unsurprising that older children have worse HRQL than younger children.

**Table 1:** Linear regression analyses for children with specialist-diagnosed food allergy, by age group (n = 63)

HRQL	Unadjusted			Model 1^†^			Model 2^‡^		
	B	95% CI	*p* value	B	95% CI	*p* value	B	95% CI	*p* value
Overall									
0–5 years	Ref			Ref			Ref		
6–12 years	*1.21*	*0.61–1.82*	*<0.001*	*0.71*	*0.18–1.23*	*0.01*	*0.60*	*0.05–1.16*	*0.04*
FA									
0–5 years	Ref			Ref			Ref		
6–12 years	*1.51*	*0.93–2.09*	*<0.01*	*1.06*	*0.52–1.61*	*<0.001*	*1.09*	*0.50–1.67*	*0.001*
EI									
0–5 years	Ref			Ref			Ref		
6–12 years	*1.27*	*0.67–1.88*	*<0.001*	*0.76*	*0.21–1.31*	*0.008*	*0.66*	*0.07–1.24*	*0.03*
SDL									
0–5 years	Ref			Ref			Ref		
6–12 years	*0.94*	*0.18–1.69*	*0.02*	0.39	− 0.35–1.13	0.30	0.20	− 0.58–0.97	0.61

*95% CI* 95th percent confidence interval, *EAI* epinephrine autoinjector, *EI* emotional impact, *FA* food anxiety, *HRQL* health-related quality of life, *SDL* social and dietary limitations

^†^Adjusted for symptom severity, EAI prescription and number of concomitant allergic diseases

^‡^Adjusted for symptom severity, EAI prescription, number of concomitant allergic diseases and region

### Proxies of severity

Compared to children with less severe symptoms only, children with more severe symptoms had worse HRQL overall and across domains, in unadjusted models only (Table 2). Compared to children without an EAI prescription, those who did had worse overall HRQL (ββ = 0.57, 95% CI − 0.01–1.15, *p* = 0.05) and FA (β = 0.97, 95% CI 0.36–1.58, *p* = 0.002), but not EI or SDL. Previously, we showed that severe symptoms and EAI prescription were associated with worse overall HRQL amongst children with staple food allergy [6]. Herein, we extended these analyses to include HRQL domains, the results of which showed that this association was exclusively driven FA amongst those with an EAI prescription. The questions that contribute to this domain capture the worry, fear and need for caution and concern when eating, including eating unfamiliar foods in unfamiliar places.

**Table 2:** Linear regression analyses for children with specialist-diagnosed food allergy, by proxies of severity (n = 59)

HRQL	Unadjusted			Model 1^†^			Model 2^‡^		
	B	95% CI	*p* value	B	95% CI	*p* value	B	95% CI	*p* value
A. Symptom severity									
Overall									
Less^§^	Ref			Ref			Ref		
More^¶^	*0.95*	*0.29–1.61*	*0.005*	0.46	− 0.13–1.05	0.12	0.51	− 0.09–1.10	0.09
FA									
Less^§^	Ref			Ref			Ref		
More^¶^	*0.89*	*0.20–1.59*	*0.01*	0.28	0.33–0.89	0.37	0.27	− 0.36–0.89	0.39
EI									
Less^§^	Ref			Ref			Ref		
More^¶^	*1.01*	*0.34–1.67*	*0.004*	0.55	− 0.07–1.16	0.81	0.59	− 0.03–1.22	0.06
SDL									
Less^§^	Ref			Ref			Ref		
More¶	*0.96*	*0.13–1.78*	*0.02*	0.48	− 0.34–1.31	0.24	0.57	− 0.25–1.39	0.17
B. EAI prescription									
Overall									
No	Ref			Ref			Ref		
Yes	*0.99*	*0.44–1.55*	*0.001*	*0.70*	*0.17–1.23*	*0.01*	*0.57*	*−* *0.01–1.15*	*0.05*
FA									
No	Ref			Ref			Ref		
Yes	*1.22*	*0.62–1.81*	*<0.001*	*0.94*	*0.39–1.50*	*0.001*	*0.97*	*0.36–1.58*	*0.002*
EI									
No	Ref			Ref			Ref		
Yes	*1.04*	*0.44–1.63*	*0.001*	*0.68*	*0.13–1.24*	*0.02*	0.55	− 0.06–1.16	0.08
SDL									
No	Ref			Ref			Ref		
Yes	*0.78*	*0.07–1.50*	*0.03*	0.53	− 0.21–1.28	0.16	0.29	− 0.51–1.10	0.47
C. Number of concomitant allergic disease									
Overall									
0–2	Ref			Ref			Ref		
3	*0.93*	*0.29–1.57*	*0.005*	0.41	− 0.13–0.95	0.13	0.42	− 0.12–0.95	0.13
FA									
0–2	Ref			Ref			Ref		
3	*0.81*	*0.13–1.49*	*0.02*	0.24	− 0.32–0.79	0.40	0.23	− 0.33–0.80	0.41
EI									
0–2	Ref			Ref			Ref		
3	*0.93*	*0.27–1.58*	*0.006*	0.39	− 0.18–0.95	0.17	0.39	− 0.17–0.96	0.17
SDL									
0–2	Ref			Ref			Ref		
3	*1.07*	*0.31–1.82*	*0.006*	0.63	− 0.13–1.38	0.10	0.64	− 0.11–1.38	0.09

*95% CI* 95th percent confidence interval, *EAI* epinephrine autoinjector, *EI* emotional impact, *FA* food anxiety, *HRQL* health-related quality of life, *SDL* social and dietary limitations

^†^Adjusted for age, EAI prescription and number of concomitant allergic diseases

^‡^Adjusted for age, EAI prescription, number of concomitant allergic diseases and region

^§^Less severe symptoms: skin, oral, gastroenteritis and/or rhinoconjunctivitis

^¶^More severe symptoms: respiratory and/or cardiovascular

Like other authors, we found no differences in HRQL when comparing 3 vs. 0–2 concomitant allergic diseases in models adjusted for EAI prescription and symptom severity [4, 8]. More severe food allergy may reduce any effects of concomitant allergic diseases on HRQL, potentially because food allergy demands constant vigilance during meals and social events, whereas concomitant allergic diseases do not necessarily demand continual awareness. At the same time, children with food allergy and concomitant asthma are at greater risk for severe allergic reactions, including anaphylaxis. Thus, concomitant allergic diseases should not be dismissed entirely when evaluating food allergy-related HRQL. Most of our study participants had at least one concomitant allergic disease, which may have diluted the impact of these diseases.

Recently, food allergy has been linked with anxiety [10]. The FAQLQ-PF is not designed or intended to identify this condition. However, we found that the domains EI and FA, but not SDL, were more often significantly worse for children with more severe disease. These findings provide additional evidence that the emotional challenges of food allergy are more impactful than the requisite behavioural changes.

## Conclusion

Older children and those with severe food allergy have poor HRQL, particularly within the domains FA and EI. These findings suggest that the emotional challenges of food allergy are more impactful than the requisite behavioural changes, particularly as school-aged children reach an age where they start assume greater self-management.

## Additional files

**Additional file 1: Table S1.** Description of characteristics of the three HRQL domains. **Table S2**. Characteristics of children aged 0–12 years with specialist-diagnosed food allergy. **Table S3**. Overall and domain-specific HRQL mean scores in the entire study population (n = 63). **Table S4.** Disease severity and overall HRQL according to participant background, symptoms of food allergy, type of food allergy and presence of concomitant allergic diseases in children with specialist-diagnosed food allergy, aged 0–12 years.

**Additional file 2: Figure S1.** Enrolment of children aged 0-12 years with specialist-diagnosed food allergy, recruited from allergology clinics.

## Abbreviations

EAI: epinephrine auto-injector
EI: emotional impact (a domain within the FAQLQ-PF)
FA: food anxiety (a domain within the FAQLQ-PF)
FAQLQ-PF: food allergy quality of life questionnaire-parent form
FoodHE II: food allergy and health economics study phase II
HRQL: health-related quality of life
IgE: immunoglobulin E
MCID: minimal clinically important difference
OFC: oral food challenge
SDL: social and dietary limitations (a domain within the FAQLQ-PF)
sIgE: allergen-specific IgE antibodies
95% CI: 95th percent confidence interval
