# Supplementary material for: Health-related quality of life worsens by school age amongst children with food allergy
Source: Clin Transl Allergy. 2019 Feb 7;9:10. doi: 10.1186/s13601-019-0244-0 (PMC6366088; doi:10.1186/s13601-019-0244-0)
Supplement: Supplementary file 1 — Additional file 1: Table S1. Description of characteristics of the three HRQL domains. Table S2. Characteristics of children aged 0–12 years with specialist-diagnosed food allergy. Table S3. Overall and domain-specific HRQL mean scores in the entire study population (n = 63). Table S4. Disease severity and overall HRQL according to participant background, symptoms of food allergy, type of food allergy and presence of concomitant allergic diseases in children with specialist-diagnosed food allergy, aged 0–12 years. [file 13601_2019_244_MOESM1_ESM.docx]

Table S1: Description of characteristics of the three HRQL domains

| HRLQ Element | Description |
| --- | --- |
| Overall HRQL | Mean score of all three HRQL domains (EI, FA, SDL) |
| EI | Feeling discouragement, disappointment, lack of control and worrying about the future, due to food allergy |
| FA | Worrying about food, eating out, or poor labelling on products, due to food allergy |
| SDL | Limitations in diet or social life, not being able to attend social activities, due to food allergy |

Abbreviations: EI, Emotional Impact, FA, Food Anxiety, HRQL, Health-Related Quality of Life, SDL, Social and Dietary Limitations

Table S2: Characteristics of children aged 0-12 years with specialist-diagnosed food allergy

|  | | | | | N | % |  |
| --- | --- | --- | --- | --- | --- | --- | --- |
| Gender | | Boy | | | 36 | 57.1 |  |
| Girl | | | | | 27 | 42.9 |  |
| Age | | 0-4y | | | 25 | 39.7 |  |
| 5-7y | | | | | 23 | 36.5 |  |
| 8-12y | | | | | 15 | 23.8 |  |
| Food allergy¶ | | Hen’s egg | | | 40 | 63.5 |  |
| Tree nut | | | | | 32 | 50.8 |  |
| Peanut | | | | | 28 | 44.4 |  |
| Cow’s milk | | | | | 27 | 42.9 |  |
| Fruit | | | | | 11 | 15.9 |  |
| Wheat | | | | | 9 | 14.3 |  |
| Shellfish | | | | | 8 | 12.7 |  |
| Fish | | | | | 7 | 11.1 |  |
| Soy | | | | | 7 | 11.1 |  |
| Vegetables | | | | | 7 | 11.1 |  |
| Sesame seeds | | | | | 2 | 3.2 |  |
| Other | | | | | 8 | 12.7 |  |
| Food allergy ¶ by age group | | | | | 0-5 y | | |
| Hen’s egg | | | | | 25 | 78.1 |  |
| Tree nut | | | | | 11 | 34.4 |  |
| Peanut | | | | | 7 | 21.9 |  |
| Cow’s milk | | | | | 15 | 46.9 |  |
|  | | | | | 6-12 y | |  |
| Hen’s egg | | | | | 15 | 48.4 |  |
| Tree nut | | | | | 21 | 67.7 |  |
| Peanut | | | | | 21 | 67.7 |  |
| Cow’s milk | | | | | 12 | 38.7 |  |
| Number of foods allergies | | | 1 | | 15 | 23.8 |  |
| 2-3 | | | | | 29 | 46.0 |  |
| ≥4 | | | | | 19 | 30.2 |  |
| Symptoms¶† | | Skin‡ | | | 56 | 94.9 |  |
| Respiratory§ | | | | | 39 | 66.1 |  |
| Oral‡ | | | | | 38 | 64.4 |  |
| Gastrointestinal‡ | | | | | 35 | 59.3 |  |
| Rhinoconjunctivitis‡ | | | | | 35 | 59.3 |  |
| Cardiovascular§ | | | | | 10 | 16.9 |  |
| Anaphylaxis† | Yes | | | | 25 | 43.1 |  |
| No | | | | | 33 | 56.9 |  |
| Epinephrine auto injector prescription† | | | Yes | | 24 | 45.3 |  |
| No | | | | | 29 | 54.7 |  |
| Number of concomitant allergic diseases†* | | | | 0 | 10 | 16.9 |  |
| 1-2 | | | | | 23 | 39.0 |  |
| 3 | | | | | 26 | 44.1 |  |

**†**Parent-reported
‡ Less severe symptoms
§ More severe symptoms

¶Not mutually exclusive
*Asthma, rhinitis, atopic dermatitis

Table S3: Overall and domain-specific HRQL mean scores in the entire study population (n=63)

| HRQL | Mean | SD | p-value* |
| --- | --- | --- | --- |
| Overall | 2.65 | 1.32 | Ref |
| FA | 2.48 | 1.38 | 0.48 |
| EI | 2.56 | 1.35 | 0.70 |
| SDL | 2.89 | 1.56 | 0.35 |

*****Compared to overall HRQL.

Abbreviations: *EI*, Emotional Impact, *FA*, Food Anxiety, *HRQL*, Health-Related Quality of Life, *SD*, Standard Deviation, *SDL*, Social and Dietary Limitations

Table S4: Disease severity and overall HRQL according to participant background, symptoms of food allergy, type of food allergy and presence of concomitant allergic diseases in children with specialist-diagnosed food allergy, aged 0-12 years.

|  |  | N | Mean | p-value |
| --- | --- | --- | --- | --- |
| Age | 0-5 years | 32 | 2.06 | <0.001 |
|  | 6-12 years | 31 | 3.27 |  |
| Gender | Boy | 36 | 2.83 | 0.24 |
|  | Girl | 27 | 2.42 |  |
| Income† | Lower | 34 | 2.48 | 0.33 |
|  | Higher | 21 | 2.84 |  |
| Symptom severity‡ | Less severe | 19 | 1.98 | 0.01 |
|  | More severe | 40 | 2.93 |  |
| Number of symptoms | 0-3 | 8 | 1.53 | 0.01 |
|  | ≥4 | 51 | 2.80 |  |
| Previous anaphylaxis | No | 33 | 2.10 | <0.001 |
|  | Yes | 25 | 3.30 |  |
| EAI prescription | No | 29 | 2.13 | 0.001 |
|  | Yes | 24 | 3.12 |  |
| Number of food allergies | 1-2 | 34 | 2.08 | <0.001 |
|  | ≥3 | 29 | 3.33 |  |
| Number of allergic disorders | 0-2 | 33 | 2.27 | 0.02 |
|  | 3 | 30 | 3.07 |  |

† The cut-off for income (total household income after taxes) between the two groups “lower” and “higher” was set at the mean of 5021 € per month.
‡ Less severe symptoms: skin, oral, gastroenteritis and/or rhinoconjunctivitis. More severe symptoms: respiratory and/or cardiovascular.

Abbreviations: *EAI*, Epinephrin
