# Supplementary material for: Health-related quality of life worsens by school age amongst children with food allergy
Source: Clin Transl Allergy. 2019 Feb 7;9:10. doi: 10.1186/s13601-019-0244-0 (PMC6366088; doi:10.1186/s13601-019-0244-0)
Supplement: Supplementary file 2 — Additional file 2: Figure S1. Enrolment of children aged 0-12 years with specialist-diagnosed food allergy, recruited from allergology clinics. [file 13601_2019_244_MOESM2_ESM.docx]

Figure S1: Enrolment of children aged 0-12 years with specialist-diagnosed food allergy, recruited from allergology clinics
